# In Vitro Antileishmanial Activity and Cytotoxicity Evaluation of 2-(Thiophen-2-ylmethyl)quinazolin-4(3*H*)-one Derivatives Against *Leishmania tropica* and *Leishmania infantum*

**DOI:** 10.3390/ph19101516

**Published:** 2026-09-24

**Authors:** Atilla Karabağ, Baycan Mor

**Affiliations:** 1 Department of Biology, Graduate School of Natural and Applied Sciences, Kafkas University, 36100 Kars, Türkiye; atillakarabag@icloud.com; 2 Department of Molecular Biology and Genetics, Faculty of Sciences and Letters, Kafkas University, 36100 Kars, Türkiye

**Keywords:** *Leishmania tropica*, *Leishmania infantum*, quinazolin-4(3*H*)-one, antileishmanial activity, selectivity index, molecular docking

## Abstract

**Background/Objectives:** Leishmaniasis is a neglected tropical disease and the toxicity, limited efficacy and emerging drug resistance of current therapies underscore the urgent need for new antileishmanial agents. This study aimed to experimentally assess the in vitro antileishmanial activity, cytotoxicity and selectivity of quinazolin-4(3*H*)-one derivatives against *Leishmania tropica* and *Leishmania infantum* and to computationally investigate the potential interaction of compound E9 with trypanothione reductase. **Methods:** The in vitro antileishmanial activities of sixteen quinazolin-4(3*H*)-one derivatives (E1-E16) against the promastigote forms of *L. tropica* and *L. infantum* were evaluated. Additionally, the most active compounds were subjected to an evaluation of their cytotoxicity in human umbilical vein endothelial cells (HUVECs) and their selectivity indices (SIs) were subsequently calculated. Molecular docking and molecular mechanics/generalized Born surface area (MM/GBSA) analyses were performed for compound E9 against *L. infantum* trypanothione reductase. **Results:** Compounds E2, E9 and E11 showed activity against *L. tropica*, with half-maximal inhibitory concentration (IC_50_) values of 23.2, 22.4 and 76.0 μM, respectively. In contrast, compounds E2, E3, E9 and E11 were active against *L. infantum*, with IC_50_ values of 38.1, 69.7, 28.2 and 86.7 μM, respectively, suggesting species-dependent activity. Furthermore, among the tested compounds, E2 showed the most favorable selectivity profile against *L. tropica* and *L. infantum*, with SI values of 8.8 and 5.4, respectively. Moreover, molecular docking of E9 produced a docking score of −8.070 kcal/mol, with MM/GBSA analysis indicating a binding free energy of −82.44 kcal/mol. **Conclusions:** These findings support further investigation of quinazolin-4(3*H*)-one derivatives as potential antileishmanial agents, with E2 emerging as the most selective compound. Furthermore, they suggest that the biological activity of this compound series may differ among *Leishmania* species. The molecular modeling findings suggest a potential interaction between E9 and trypanothione reductase, supporting the need for further evaluation of these compounds in intracellular amastigote models and subsequent in vivo studies.

## 1. Introduction

Leishmaniasis is a neglected tropical disease caused by protozoan parasites of the genus *Leishmania* and transmitted by vectors, representing a major global public health concern. The disease mostly affects populations in tropical and subtropical regions because low socioeconomic conditions and limited access to healthcare services in these areas facilitate its spread, making effective control more difficult [1]. Over one billion people live in regions where leishmaniasis is prevalent, placing them at risk of infection. Moreover, more than one million new cases of cutaneous leishmaniasis (CL), along with approximately 30,000 new cases of visceral leishmaniasis (VL), are estimated to occur each year [2]. In addition to its effects on health, leishmaniasis intensifies poverty and social vulnerability, disproportionately affecting disadvantaged communities [3,4,5]. Clinically, leishmaniasis is classified into three principal forms: CL, VL and mucocutaneous leishmaniasis (MCL). CL is the most prevalent form and is characterized by ulcerative skin lesions that may lead to permanent scarring and significant psychosocial consequences [2,6]. In contrast, VL is the most severe clinical form and can lead to death when left untreated [2,7].

Among the etiological agents of leishmaniasis, *Leishmania tropica* and *Leishmania infantum* are of particular relevance from both clinical and epidemiological perspectives. *L. tropica* is a major causative agent of CL, especially in the Middle East, with increasing incidences reported in conflict-affected regions [6,8]. However, *L. infantum* is the main etiological agent responsible for VL across Europe and the Mediterranean basin and effective disease control is complicated by the zoonotic transmission cycle, in which domestic dogs act as the main reservoir hosts [3,7]. Furthermore, HIV co-infection in endemic areas is associated with greater disease severity and increased challenges in clinical management [6,9].

The current treatment primarily consists of pentavalent antimonials, amphotericin B (including its liposomal formulation) and miltefosine. However, these drugs have significant limitations. While the clinical utility of pentavalent antimonials has declined in some endemic areas owing to the development of drug resistance accompanied by serious side effects [10,11,12], liposomal amphotericin B is regarded as a safer therapeutic option; however, its substantial cost restricts its accessibility and utilization in economically disadvantaged regions [13,14]. Furthermore, miltefosine, currently the only available oral treatment, has an even more restricted role due to gastrointestinal toxicity, emerging drug resistance and the prolonged duration of therapy [15,16]. These limitations necessitate the development of safer, more effective, affordable and shorter-duration treatment alternatives [17,18].

Heterocyclic compounds, especially those containing nitrogen, exhibit a wide range of biological activities and hold significant pharmaceutical potential. Therefore, they have long played a significant role in medicinal chemistry. Of these, quinazolinones constitute a particularly outstanding and extensively investigated subclass, owing to their diverse therapeutic properties, including antileishmanial, anticancer and antimicrobial effects [19,20,21,22]. Various methodologies, especially cyclization and condensation reactions, can be employed to synthesize these scaffolds [23]. In addition, their structural flexibility facilitates the optimization of pharmacological profiles through simple chemical modifications [20,24]. Structural modifications of the quinazolinone scaffold have been reported to influence its biological activity [20,25,26], highlighting the importance of this scaffold in rational drug design.

In a previous study, it was concluded that 2-(thiophen-2-ylmethyl)quinazolin-4(3*H*)-one Schiff bases demonstrated promising in vitro antileishmanial activity against *Leishmania major* and *Leishmania donovani* promastigotes [22]. However, their activity against other clinically relevant *Leishmania* species has not been characterized. The present study extends the evaluation of this same compound series to *L. tropica* and *L. infantum*, thereby providing new comparative information on species-dependent antileishmanial activity and selectivity within the series. In Türkiye, cutaneous leishmaniasis is mostly caused by *L. tropica*, while *L. infantum* is the major causative agent of visceral leishmaniasis. Accordingly, the major objectives of the present study were to evaluate the in vitro antileishmanial activity of these 2-(thiophen-2-ylmethyl)quinazolin-4(3*H*)-one Schiff base derivatives against *L. tropica* and *L. infantum*, to assess the cytotoxicity and selectivity profiles of the active derivatives, and to investigate the potential molecular interactions of the most active compound with the target protein through molecular docking analysis.

## 2. Results

### 2.1. In Vitro Antileishmanial Activity and Cytotoxicity Assay

Of the sixteen compounds evaluated, only E2, E3, E9 and E11 exhibited half-maximal inhibitory concentration (IC_50_) values below 100 µM against *L. infantum* promastigotes after 24 h of treatment, with IC_50_ values of 38.1, 69.7, 28.2 and 86.7 µM, respectively. Additionally, the reference drug miltefosine exhibited an IC_50_ value of 33.8 µM against *L. infantum* (Figure 1A–C).

Similarly, compounds E2, E9 and E11 also demonstrated IC_50_ values below 100 µM against *L. tropica* following 24 h of treatment, with IC_50_ values of 23.2, 22.4 and 76.0 µM, respectively, while miltefosine exhibited an IC_50_ value of 21.8 µM (Figure 1D–F).

The cytotoxic effects of the selected compounds (E2, E3, E9 and E11) on human umbilical vein endothelial cells (HUVECs) were evaluated following 24 h of exposure. The IC_50_ values of compounds E2, E3, E9 and E11 were determined to be 205.3, 84.6, 17.2 and 133.1 µM, respectively, whereas the IC_50_ value of the reference drug miltefosine was 7.6 µM (Figure 2).

Moreover, the selectivity indices (SIs) of the selected compounds were calculated to provide a more comprehensive assessment of their cytotoxic and antileishmanial activities (Table 1). Of the compounds tested, E2 showed the highest selectivity index (SI = 5.4) against *L. infantum*. This finding reflects its low cytotoxicity against HUVECs (IC_50_ = 205.3 µM) and positive antileishmanial activity (IC_50_ = 38.1 µM). In contrast, E11 (SI = 1.5) and E3 (SI = 1.2) displayed lower selectivity, whereas E9 (SI = 0.6) and the reference drug miltefosine (SI = 0.2) demonstrated the lowest selectivity indices.

With respect to *L. tropica*, E2 again demonstrated the highest selectivity index (SI = 8.8); E11 showed moderate selectivity (SI = 1.8), whereas E9 (SI = 0.8) and the reference drug miltefosine (SI = 0.3) exhibited comparatively lower selectivity indices. When considered together, these findings suggest that E2 demonstrated the highest selectivity among the tested compounds against *Leishmania* species, with greater selectivity toward *L. tropica* than *L. infantum*.

### 2.2. Molecular Docking Analysis

Among the evaluated compounds, E9 exhibited the lowest IC_50_ values against both *L. infantum* and *L. tropica* and was therefore selected for molecular docking analysis. The selection of E9 was based primarily on its overall antiparasitic potency against both *Leishmania* species, rather than on its selectivity profile. The docking analysis was intended to investigate the potential binding mode and molecular interactions of the most active compound with Leishmania trypanothione reductase (TR), which was considered a potential molecular target of the studied compounds. Thus, the docking results were used to provide a molecular-level interpretation of the biological activity of the most potent derivative and were not intended to directly explain species or host-cell selectivity.

Compound E9 exhibited a docking score of −8.070 kcal/mol in the molecular docking analysis against *L. infantum* trypanothione reductase (TR; PDB ID: 2JK6) [27,28]. Subsequent MM/GBSA calculations yielded a predicted binding free-energy estimate of −82.44 kcal/mol, indicating favorable computational binding energetics. Furthermore, the predicted binding mode of E9 within the TR active site suggested the formation of multiple non-covalent interactions (Figure 3). Specifically, the quinazolinone carbonyl oxygen was predicted to form a hydrogen bond with Lys60 at a distance of 1.83 Å, while the morpholine oxygen was predicted to form a second hydrogen bond with Gln439 at a distance of 2.31 Å. In addition, the quinazolinone core and thiophene ring of E9 were predicted to engage in cation–π interactions with the positively charged guanidinium group of Arg287, with corresponding centroid–cation distances of 4.59 and 3.87 Å, respectively. Both distances fall within the ≤6 Å cutoff commonly used for the identification of geometrically plausible cation–π interactions in protein–ligand complexes [29,30]. These predicted contacts may contribute to the positioning of the aromatic moieties of E9 within the TR binding pocket. Nevertheless, as these interactions were identified from the docking geometry, they should be regarded as putative cation–π contacts rather than experimentally established interactions. Collectively, these computational findings suggest that E9 may establish favorable interactions within the TR active site; however, they should be regarded as predictive rather than definitive evidence of stable binding or TR inhibition.

To assess the reliability of the applied molecular docking protocol, a redocking validation was performed using the crystallographic structure of TR. The crystal structure was determined by X-ray diffraction at a resolution of 2.95 Å and contains the native flavin adenine dinucleotide (FAD) cofactor within the enzyme binding site. The co-crystallized FAD ligand was removed from the binding site and subsequently redocked using the same protocol employed for the investigated compound.

The superposition of the experimentally determined and redocked FAD conformations resulted in an RMSD value of 0.9427 Å, indicating a high degree of agreement between the predicted and experimentally observed binding modes (Figure 4). This RMSD value, being well below the commonly accepted 2.0 Å criterion, demonstrates that the docking protocol can reliably reproduce the experimentally determined ligand orientation [31,32]. As shown in Figure 4, the native FAD conformation is depicted in green, whereas the redocked FAD is shown in magenta. Nitrogen and oxygen atoms are represented in blue and red, respectively. The close structural overlap between the two conformations further supports the validity of the docking methodology used in the present study.

It should be emphasized that the molecular docking findings presented herein do not establish TR as the molecular target responsible for the antileishmanial activity of E9. In the absence of an experimental TR inhibition assay, the predicted binding interactions should be regarded as suggestive of a potential interaction between E9 and TR rather than as direct evidence of target engagement or enzyme inhibition. Thus, the docking results provide a plausible molecular hypothesis that may contribute to the interpretation of the observed biological activity, but they do not establish a causal relationship between TR inhibition and the antileishmanial effect. Experimental biochemical studies assessing TR inhibition, together with appropriate target-validation approaches, will be required to confirm this proposed mechanism.

### 2.3. Molecular Dynamics Simulation Results

To further assess the structural stability and dynamic behavior of the E9–TR complex predicted by molecular docking, a 50 ns molecular dynamics (MD) simulation was performed. The simulation was conducted to examine the temporal stability of the docked complex and to determine whether the key interactions predicted by the docking analysis could be maintained during the simulation. The structural behavior of the protein–ligand complex was evaluated by monitoring parameters including the root-mean-square deviation (RMSD) and root-mean-square fluctuation (RMSF), together with the persistence of ligand–residue interactions throughout the simulation. The results of the 50 ns MD simulation of the E9–TR complex are presented in Figure 5.

During the simulation, the quinazolinone carbonyl oxygen maintained a hydrogen-bond interaction with Lys60 with a high occupancy of 99%, indicating that this interaction was highly persistent throughout the trajectory. The hydrogen bond between the morpholine oxygen and Gln439 was retained with an occupancy of 39%. In addition, the morpholine oxygen participated in a water-mediated hydrogen-bond interaction with Ala363, suggesting that the polar morpholine moiety remained engaged with the surrounding binding-site environment during the simulation (Figure 5a).

The Cα RMSD of the protein remained relatively low, with an average value of 1.75 Å (turquoise), whereas the ligand exhibited an average RMSD of approximately 2.0 Å (red). The overall deviation of the complex remained limited, with a reported deviation of approximately 0.4 Å (pink), indicating no pronounced structural rearrangement during the 50 ns simulation (Figure 5b). The RMSF values for both the protein Cα atoms (Figure 5c) and the ligand (Figure 5d) were approximately 0.8 Å, further indicating relatively limited fluctuations throughout the trajectory. In addition, approximately twenty protein–ligand contacts were observed during the simulation, as represented by the green vertical bars in Figure 5c. Among these interactions, Lys60 and Gln439 exhibited the highest interaction occupancies (Figure 5e). The MD trajectory indicates that E9 remained associated with the TR binding region and several of the key interactions predicted by docking, particularly the Lys60 hydrogen bond, were maintained to a substantial extent during the simulation. These findings provide additional computational support for the plausibility of the docked binding mode, while not constituting experimental confirmation of TR binding or inhibition.

### 2.4. Post-MD MM/GBSA Analysis Results

To further evaluate the energetic behavior of the E9–TR complex during the MD trajectory, a post-MD MM/GBSA analysis was performed using representative frames extracted from the 50 ns simulation [33]. A total of 21 frames, corresponding to 2.5 ns intervals from 0 to 50 ns, were subjected to Prime MM/GBSA calculations. The calculated ΔG_bind values ranged from −88.10 to −66.57 kcal/mol, with a mean binding free-energy estimate of −78.52 ± 4.29 kcal/mol. The trajectory-based mean value was comparable to the MM/GBSA value obtained from the initial docking pose (−82.44 kcal/mol), indicating that the favorable energetic profile predicted for the E9–TR complex was generally maintained across the conformational states sampled during the MD simulation.

## 3. Discussion

When considered together with a previous study evaluating the same quinazolin-4(3*H*)-one Schiff base derivatives against *L. major* and *L. donovani*, the present study provides more evidence of significant variation in drug susceptibility among *Leishmania* species. The previous study identified compounds 2 and 9, corresponding to E2 and E9 in the present study, as exhibiting the most notable antileishmanial activity against *L. major* and *L. donovani* [22]. In contrast, the activity profile observed in the present study differed between *L. tropica* and *L. infantum*. E2, E9 and E11 demonstrated the highest antileishmanial activity against *L. tropica*, while E2, E3, E9 and E11 exhibited activity against *L. infantum*. Remarkably, E3 displayed species-specific activity, showing antileishmanial activity against *L. infantum* while remaining inactive against *L. tropica*. Taken together, these findings suggest that even closely related quinazolin-4(3*H*)-one derivatives can produce distinct, species-dependent biological responses among *Leishmania* species.

This is particularly remarkable because *L. tropica* is the main causative agent of cutaneous leishmaniasis in Türkiye, while most visceral leishmaniasis cases in the country are caused by *L. infantum* [34,35,36]. Thus, assessing the same compound series against these two epidemiologically and clinically important *Leishmania* species allows for a more comprehensive evaluation of their antileishmanial potential against the species of particular relevance in Türkiye. Furthermore, the present findings are consistent with previous studies demonstrating that structural modifications of quinazolinone derivatives can substantially influence their biological activity against different *Leishmania* species [20,21].

Additionally, one of the most remarkable findings of the present study was the species-dependent variation in susceptibility to the tested compounds. While compounds E2 and E9 exhibited comparable antileishmanial activity against both *Leishmania* species, compound E3 was active only against *L. infantum* and showed no significant activity against *L. tropica*. The mechanisms underlying this species-dependent difference were not directly investigated in the present study. This difference may stem from intrinsic biological and molecular differences among *Leishmania* species. Supporting this possibility, previous studies have demonstrated substantial interspecies differences in membrane lipid composition, surface glycoconjugates, particularly lipophosphoglycan (LPG), and membrane fluidity [37,38,39]. Such structural differences may, in turn, directly influence cellular uptake, intracellular distribution and access of compounds to their molecular targets. Therefore, the species-specific activity of E3 may reflect these fundamental biological differences among *Leishmania* species.

The species-dependent differences observed in the present study may not be explained solely by variations in parasite surface architecture but may also reflect differences in fundamental metabolic and cellular processes. Redox homeostasis and parasite-specific antioxidant defense systems are essential for *Leishmania* survival and are thought to play a role in susceptibility and resistance to antileishmanial compounds [40,41,42]. Additionally, drug uptake, intracellular accumulation and efflux may be influenced by alterations in drug transport, including changes in the expression or activity of ATP-dependent transporters and other membrane transport proteins, potentially contributing to differences in drug susceptibility [18]. Taken together, these considerations suggest that the species-specific activity observed in the present study may reflect structural differences among *Leishmania* species as well as underlying biochemical and physiological factors that affect drug susceptibility.

The mechanisms underlying these species-specific differences need to be clarified. Species-specific differences in drug uptake, membrane permeability, efflux, intracellular metabolism and the structural properties or expression levels of molecular targets may underlie the observed differences in antileishmanial activity. Accordingly, further molecular investigations, including gene expression analyses, protein-level characterization and target validation, are needed to clarify the mechanisms underlying these differences. In general, these findings emphasize the importance of evaluating potential antileishmanial compounds among multiple *Leishmania* species rather than relying on a single experimental model.

Additionally, compound E2 exhibited IC_50_ values against both *L. infantum* and *L. tropica* that were similar to those of the reference agent miltefosine under the present in vitro experimental conditions. However, similarity in promastigote IC_50_ values should not be interpreted as evidence of equivalent efficacy, as pharmacokinetic properties, activity against intracellular amastigotes, mechanism of action and in vivo exposure may differ substantially. Nevertheless, the further evaluation of these compounds should consider not only their antileishmanial activity but also their cytotoxicity toward host cells, making in vitro selectivity an important parameter in their assessment [12,18].

In this context, SI analysis provided useful information on the relative in vitro selectivity of the evaluated compounds. Among the evaluated compounds, E2 exhibited the highest selectivity indices against both *L. infantum* (SI = 5.4) and *L. tropica* (SI = 8.8), indicating greater in vitro selectivity relative to the other compounds tested. However, these SI values should be interpreted cautiously and do not, by themselves, establish a favorable therapeutic window or therapeutic suitability. Notably, E2 exhibited a substantially higher IC_50_ value against HUVECs (205.3 μM) than that of the reference drug miltefosine, indicating considerably lower cytotoxicity toward host cells. In contrast, compound E9 demonstrated substantial antileishmanial activity; however, its relatively high cytotoxicity toward HUVECs resulted in low selectivity indices. Collectively, these findings highlight the well-established balance between antileishmanial potency and host-cell toxicity in antileishmanial drug discovery and underscore compound selectivity, rather than antileishmanial activity alone, as a critical determinant in the identification of promising drug candidates [15].

Furthermore, the low selectivity indices observed for miltefosine against both *Leishmania* species are consistent with its established cytotoxicity and adverse-effect profile, which restricts its clinical use [15,16]. Conversely, the higher selectivity indices of compound E2 against both species indicate a more favorable balance between antileishmanial activity and host-cell toxicity. When considered together, these findings identify E2 as a candidate for further evaluation against intracellular amastigote forms and in subsequent in vivo investigations.

Overall, E2 exhibited the most favorable in vitro activity-selectivity profile among the derivatives evaluated in this study, supporting its further evaluation in intracellular amastigote models and subsequent in vivo studies. Moreover, the species-dependent differences observed in this study underscore the importance of evaluating candidate antileishmanial compounds against multiple *Leishmania* species rather than relying on a single experimental model. When considered together, these findings support the further investigation of quinazolin-4(3*H*)-one derivatives as a scaffold for antileishmanial drug development, with E2 showing the most favorable in vitro selectivity profile among the compounds subjected to cytotoxicity evaluation.

Additionally, molecular docking analysis was performed against trypanothione reductase (TR), a therapeutically relevant enzyme in *Leishmania* species, to further investigate the potential molecular basis underlying the observed antileishmanial activity. TR plays a critical role in maintaining intracellular redox balance by catalyzing the reduction of trypanothione disulfide to trypanothione, thereby protecting the parasite from oxidative stress and supporting its survival [43,44]. Because TR is highly conserved among *Leishmania* species, including *L. infantum* and *L. tropica*, it was considered a suitable protein for the present analysis. Among the tested compounds, E9 exhibited the lowest IC_50_ values against both *L. infantum* and *L. tropica* and was therefore selected for molecular docking analysis to explore the potential molecular basis of its comparatively high antiparasitic potency. This selection was based on antileishmanial potency rather than selectivity, because the docking analysis was intended as a mechanistic investigation of the compound showing the strongest in vitro activity across both species, rather than as a prioritization of the most therapeutically favorable candidate. In contrast, E2 exhibited the most favorable in vitro selectivity profile and was therefore considered the stronger candidate for subsequent biological evaluation.

The docking and MM/GBSA analyses indicated that E9 may establish favorable interactions within the active site of TR. The predicted docking score and MM/GBSA binding free-energy estimate were accompanied by hydrogen-bonding and cation–π interactions involving residues located within the binding pocket. These computational findings provide a possible molecular explanation for the observed in vitro antileishmanial activity of E9 and suggest that interaction with TR may contribute to its biological effects. However, these findings should be interpreted cautiously, as docking and MM/GBSA calculations are predictive approaches and do not establish stable target engagement or enzyme inhibition. Experimental studies, including direct TR inhibition assays, are therefore required to determine whether TR is indeed involved in the antileishmanial mechanism of E9.

The aromatic system of compound E9 was predicted to participate in π-mediated interactions within the active site of TR. Both the quinazolinone core and the thiophene ring were predicted to form cation–π interactions with Arg287. Arg287 is located within the substrate-binding region of TR and may contribute to ligand recognition through electrostatic and π-mediated contacts. In addition, the predicted hydrogen-bonding interactions involving Lys60 and Gln439 may contribute to the positioning of E9 within the binding pocket. Taken together, these interactions indicate a plausible binding mode for E9 in the TR active site and provide a structural basis for further investigation of this interaction. Nevertheless, the predicted interactions and favorable computational energetics should not be interpreted as definitive evidence of stable binding or TR inhibition, which requires experimental validation.

The present study provides a basis for further investigation of these compounds using more advanced biological and target-validation models. In particular, studies involving intracellular amastigotes, direct TR inhibition assays and complementary target-validation approaches will help determine whether the predicted interaction with TR contributes to the antileishmanial activity of the most promising derivatives. Such studies may also provide further insight into their cellular activity and underlying mechanisms of action.

## 4. Materials and Methods

The study was designed as a sequential experimental and computational workflow comprising in vitro antileishmanial screening, cytotoxicity and selectivity assessment and molecular docking analysis. Following evaluation of the quinazolin-4(3*H*)-one Schiff base derivatives against *L. tropica* and *L. infantum*, compounds meeting the predefined activity criterion were further evaluated for cytotoxicity in HUVECs and selectivity. The overall experimental workflow is summarized in Figure 6.

### 4.1. Procurement of Study Materials

#### 4.1.1. Compounds

The quinazolin-4(3*H*)-one derivatives employed in the present study had been previously synthesized and structurally characterized. A previous study demonstrated that this series of compounds exhibited promising antileishmanial activity against *L. major* and *L. donovani* promastigotes [22]. Accordingly, the compounds were evaluated against *L. tropica* and *L. infantum* to further investigate their antileishmanial potential. All compounds were sourced from the available laboratory stocks and designated as E1–E16 throughout the study (Figure 7). The chemical names corresponding to compounds E1–E16 are provided in Table 2, while their complete synthesis and structural characterization data have been reported previously [22].

#### 4.1.2. Leishmania Isolates

*Leishmania tropica* (MHOM/AZ/1974/SAF-K27) and *Leishmania infantum* (MHOM/TN/1980/IPT1) isolates used in this study were obtained from the Parasite Bank of the Department of Parasitology, Faculty of Medicine, Manisa Celal Bayar University.

### 4.2. Culture of Leishmania tropica and Leishmania infantum Promastigotes

The *L. tropica* and *L. infantum* promastigotes, preserved by cryopreservation in liquid nitrogen at −196 °C, were recovered and rapidly thawed in a 37 °C water bath. Following thawing, the parasites were aseptically transferred into a biphasic culture system consisting of Novy-MacNeal-Nicolle (NNN) medium (HiMedia Laboratories Pvt. Ltd., Mumbai, India) with an RPMI-1640 (Gibco, Life Technologies Limited, Paisley, UK) liquid phase supplemented with 10% heat-inactivated fetal bovine serum (FBS; Gibco, Life Technologies Limited, Paisley, UK), 1.1% gentamicin (Capricorn Scientific GmbH, Ebsdorfergrund, Germany) and 1.1% penicillin-streptomycin (Gibco, Life Technologies Limited, Paisley, UK). The cultures were maintained at 27 °C under sterile conditions. Once active promastigote growth was observed, 0.5–1 mL of culture was collected from the RPMI-1640 liquid phase and transferred into sterile culture flasks containing 5 mL of fresh RPMI-1640 medium for the first passage. Promastigotes from this first passage were used for the antileishmanial activity assays. Throughout the subsequent cultivation period, 2–3 mL of fresh medium was added at two-day intervals to sustain parasite growth. Parasite proliferation was monitored by cell counting using a Thoma hemocytometer (Graticules Optics Limited, Cambridge, UK) between days 10 and 20. Under these conditions, promastigotes reached a density of approximately 1 × 10^8^ parasites/mL on day 20 [45,46].

### 4.3. In Vitro Method and Cell Viability

#### 4.3.1. Antileishmanial Activity

The in vitro antileishmanial activity of the compounds was evaluated according to the manufacturer’s protocol for the Cell Viability Detection Kit-8 (CVDK-8; EcoTech Biotechnology, Erzurum, Türkiye). Promastigote suspensions of *L. infantum* and *L. tropica* in the logarithmic phase were adjusted to a density of 1 × 10^6^ parasites per 100 µL and dispensed into sterile 96-well microplates, with each concentration evaluated in duplicate technical wells. No independent biological replicates were performed. The parasites were then exposed to the synthesized compounds at final concentrations of 100, 50, 25, 12.5, 6.25 and 3.125 µM. Miltefosine (AbMole BioScience Inc., Houston, TX, USA) was used as the reference drug at final concentrations of 100, 50, 25, 12.5, 6.25 and 3.125 µM. RPMI-1640 medium without parasites was used as the blank. Untreated parasites served as the viability control, with their absorbance defined as 100% viability. A solvent control containing DMSO (Carl Roth GmbH + Co. KG, Karlsruhe, Germany) at the same final dilution used in the compound-treated wells (1:1000) was also included. No appreciable effect of the solvent control on the CVDK-8 readout was observed relative to the untreated control. After incubation at 27 °C for 24 h, 10 µL of Cell Viability Detection Kit-8 reagent was added to each well and the plates were incubated for an additional 2 h at 37 °C. Absorbance was measured at 450 nm using a Multiskan SkyHigh Microplate Spectrophotometer (Thermo Scientific, Singapore). Cell viability was calculated by normalizing the absorbance values to those of the untreated control group, which was assumed to represent 100% viability [22]. Because the CVDK-8 assay is based on cellular metabolic activity, the absorbance signal was interpreted as an indirect measure of the viability of metabolically active promastigotes rather than as a direct measurement of parasite number.

#### 4.3.2. Cytotoxicity

Human umbilical vein endothelial cells (HUVECs; ATCC, Manassas, VA, USA) were employed to evaluate cytotoxicity. Compounds E2, E3, E9, and E11 were selected for cytotoxicity evaluation because they yielded quantifiable IC_50_ values below the predefined operational screening threshold of 100 µM against at least one of the two *Leishmania* species tested. The remaining derivatives did not meet this screening criterion and were therefore not advanced to cytotoxicity and selectivity analyses. HUVECs were cultured in Dulbecco’s Modified Eagle Medium (DMEM; Gibco, Life Technologies, Paisley, UK) supplemented with 10% FBS and 1% penicillin–streptomycin in a humidified incubator at 37 °C with 5% CO_2_. At approximately 70% confluence, the cells were detached using 0.25% trypsin (Gibco, Life Technologies, Paisley, UK) and seeded into sterile 96-well microplates at a density of 3 × 10^3^ cells per well. After 24 h of incubation, the culture medium was aspirated and replaced with fresh medium containing compounds E2, E3, E9, and E11 tested at final concentrations of 200, 100, 50, 25, 12.5, 6.25, and 3.125 µM. Miltefosine was used as the reference drug at final concentrations of 100, 50, 25, 12.5, 6.25 and 3.125 µM. All treatments were performed in duplicate technical wells and no independent biological replicates were performed. Following an additional 24 h incubation at 37 °C, 10 µL of Cell Viability Detection Kit-8 (CVDK-8; Ecotech, Erzurum, Türkiye) reagent was added to each well and the plates were incubated for an additional 2 h at 37 °C. Absorbance was measured at 450 nm using a microplate reader (Thermo Scientific, Waltham, MA, USA). Cell viability was determined by normalizing the absorbance values to those of the untreated control group, which was assumed to represent 100% viability [22].

#### 4.3.3. IC_50_ Calculations

Absorbance values obtained from the cell viability assays were normalized to the untreated control group, which was defined as representing 100% cell viability, and expressed as percentages of cell viability. Statistical analyses were performed using GraphPad Prism version 11.0 (GraphPad Software, Boston, MA, USA). IC_50_ values were determined via nonlinear regression using a variable-slope four-parameter logistic (4PL) dose–response model. For consistency and comparability with our previous evaluation of the same compound series against *L. major* and *L. donovani* [22], an IC_50_ value of 100 µM was retained as an operational screening threshold. The selectivity index (SI) for each compound was calculated as the ratio of its IC_50_ value in HUVECs to its IC_50_ value against the respective *Leishmania* species, according to the following equation: SI = IC_50_ (HUVEC)/IC_50_ (*Leishmania*). Higher SI values indicate greater selectivity toward the parasite relative to the host cells [47]. Duplicate measurements were performed and the results are presented as mean ± standard deviation (SD). Dose–response curves were generated using the same software.

### 4.4. Molecular Docking

Molecular docking studies were performed using Schrödinger Molecular Modeling Software (release 2025-1) with the Maestro interface (version 14.3). The three-dimensional crystallographic structure of trypanothione reductase from *L. infantum* (Protein Data Bank (PDB) ID: 2JK6) was retrieved from the Protein Data Bank and prepared using the Protein Preparation Wizard according to previously reported protocols [48]. The protein structure was processed by assigning appropriate bond orders and formal charges, adding missing hydrogen atoms, optimizing the hydrogen-bonding network and performing restrained energy minimization using the OPLS4 force field.

The ligand structures were prepared using the LigPrep workflow with the OPLS4 force field. Possible protonation and tautomeric states were generated under near-physiological pH conditions and the resulting structures were energy-minimized prior to docking. The receptor binding site was defined based on the position of the co-crystallized ligand in the 2JK6 crystal structure. Accordingly, the receptor grid was centered on the centroid of the co-crystallized ligand, with the outer grid-box dimensions set to 25 × 25 × 25 Å to encompass the ligand-binding cavity and surrounding residues.

Because ligand binding may induce local conformational rearrangements within the receptor, an Induced Fit Docking (IFD) protocol was employed [49,50]. In the initial docking stage, the prepared ligands were docked using Glide XP (extra precision) and up to 20 poses were generated for each ligand. The receptor residues surrounding the initially generated ligand poses were subsequently subjected to conformational refinement using Prime. The refined receptor conformations were then used for a second Glide XP docking step. The resulting poses were ranked according to the IFD and Glide docking scores and the most favorable and structurally consistent poses were selected for subsequent interaction analysis. The same standardized IFD protocol was applied to all investigated ligands to ensure consistency in the comparative evaluation.

For the selected protein–ligand complexes, Prime molecular mechanics/generalized Born surface area (MM/GBSA) calculations were subsequently performed using the OPLS4 force field and the VSGB implicit-solvent model. The binding free-energy estimate (ΔG_bind) was calculated according to the relationship ΔG_bind = G_complex − (G_protein + G_ligand), where G_complex, G_protein and G_ligand represent the calculated free energies of the protein–ligand complex, the unbound protein and the isolated ligand, respectively [51]. The resulting ΔG_bind values were used as computational estimates to assess the relative energetic favorability of the predicted protein–ligand complexes. These values were not interpreted as experimentally measured binding affinities, given the methodological and model-dependent nature of MM/GBSA calculations.

### 4.5. Molecular Dynamics Simulations

Molecular dynamics (MD) simulations were performed using the Desmond simulation package (Release 2024-4) to examine the conformational stability of the selected protein–ligand complexes under dynamic conditions. Each system was embedded in an orthorhombic simulation box and solvated with explicit TIP4P water molecules. Appropriate counterions were introduced to neutralize the systems and a physiological salt concentration of 0.15 M NaCl was added. All simulations were conducted using the OPLS4 force field. Before the production run, the systems underwent energy minimization to eliminate unfavorable steric contacts, followed by the default Desmond relaxation protocol consisting of sequential constant particle number, volume, and temperature (NVT) and constant particle number, pressure, and temperature (NPT) equilibration steps. Subsequently, a 50 ns production simulation was carried out at 300 K and 1.01325 bar. Temperature and pressure were controlled using the Nosé–Hoover thermostat and the Martyna–Tobias–Klein barostat, respectively. The dynamic stability of the complexes was assessed by analyzing the root-mean-square deviation (RMSD) and root-mean-square fluctuation (RMSF) throughout the simulation, providing information on the overall structural stability of the complexes and the flexibility of individual protein residues [52,53].

### 4.6. Post-MD MM/GBSA Analysis

To further evaluate the energetic stability of the E9–TR complex, a post-MD MM/GBSA analysis was performed using the trajectory obtained from the 50 ns molecular dynamics simulation. Following completion of the simulation, 1000 trajectory snapshots were extracted from the production trajectory. Twenty-one representative frames were subsequently selected at 2.5 ns intervals and subjected to Prime MM/GBSA calculations. For each selected frame, the binding free energy (ΔG_bind) was estimated using the OPLS4 force field and VSGB implicit-solvent model. The resulting ΔG_bind values were averaged over the 21 representative frames to obtain the mean binding free-energy estimate for the E9–TR complex. This trajectory-based MM/GBSA analysis was used to provide an additional assessment of the energetic favorability of the E9–TR complex based on its conformational ensemble during the MD simulation [54,55].

## 5. Conclusions

This study demonstrated species-dependent in vitro antileishmanial activity of 2-(thiophen-2-ylmethyl)quinazolin-4(3*H*)-one derivatives against *L. tropica* and *L. infantum*. Among the compounds subjected to cytotoxicity testing, E2 exhibited the most favorable selectivity profile, whereas E9 showed the highest antileishmanial potency against both species. Molecular docking, MD simulation, and MM/GBSA analyses suggested a potentially favorable interaction between E9 and *L. infantum* trypanothione reductase; however, these computational findings are hypothesis-generating and do not establish TR as the molecular target of E9. Further studies using intracellular amastigote models, direct target-validation approaches, and in vivo experiments are required to clarify the biological relevance and therapeutic potential of these compounds.

## Figures and Tables

**Figure 1:**
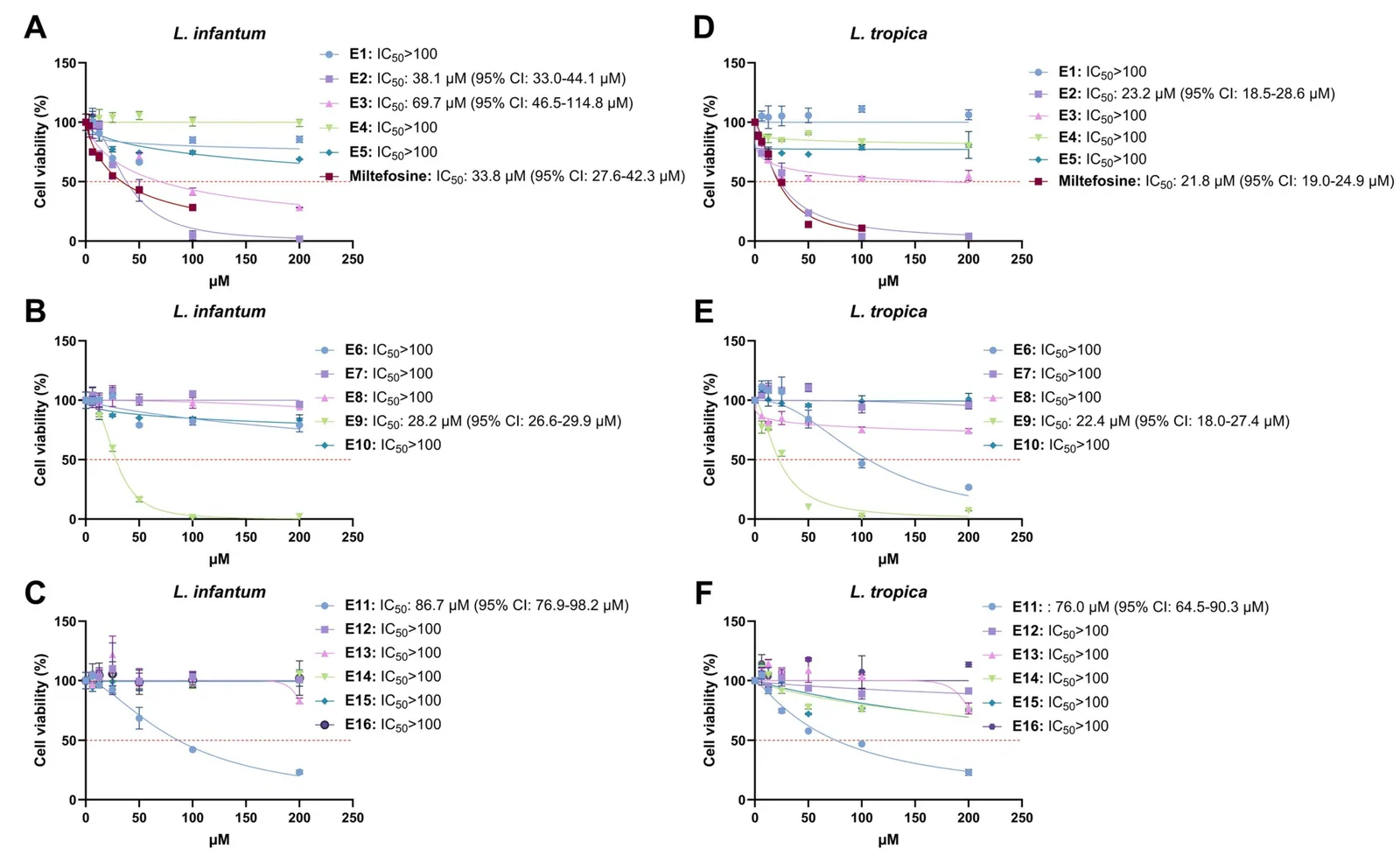
In vitro antileishmanial activity of compounds E1–E16 against *L. infantum* (**A**–**C**) and *L. tropica* (**D**–**F**). Compounds were tested at final concentrations of 3.125–100 µM. Data are presented as mean ± standard deviation from duplicate measurements (n = 2). Half-maximal inhibitory concentration (IC_50_) values were determined by nonlinear regression using a variable-slope four-parameter logistic (4PL) dose–response model.

**Figure 2:**
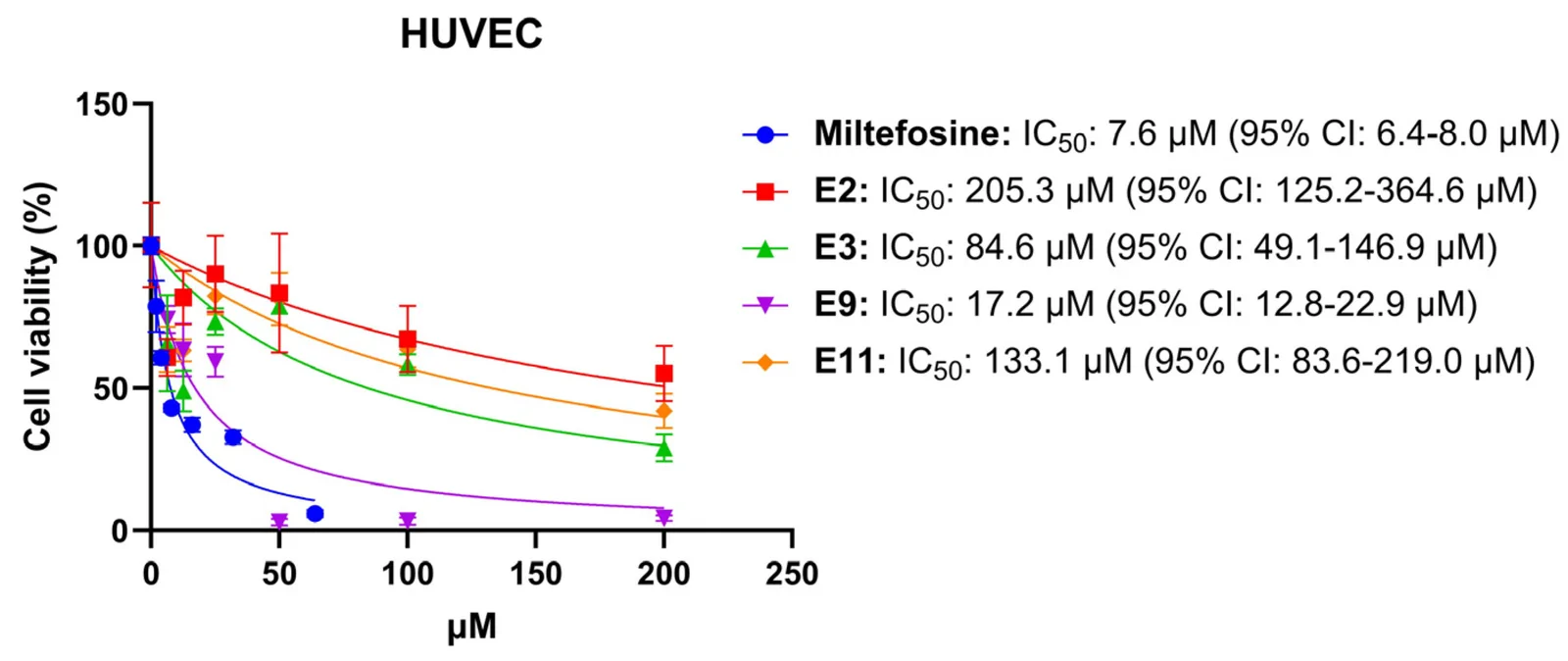
Cytotoxic effects of compounds E2, E3, E9 and E11 and the reference drug miltefosine on human umbilical vein endothelial cells (HUVECs). Data are presented as mean ± standard deviation from duplicate technical measurements (n = 2). Half-maximal inhibitory concentration (IC_50_) values were determined by nonlinear regression using a variable-slope four-parameter logistic (4PL) dose–response model.

**Figure 3:**
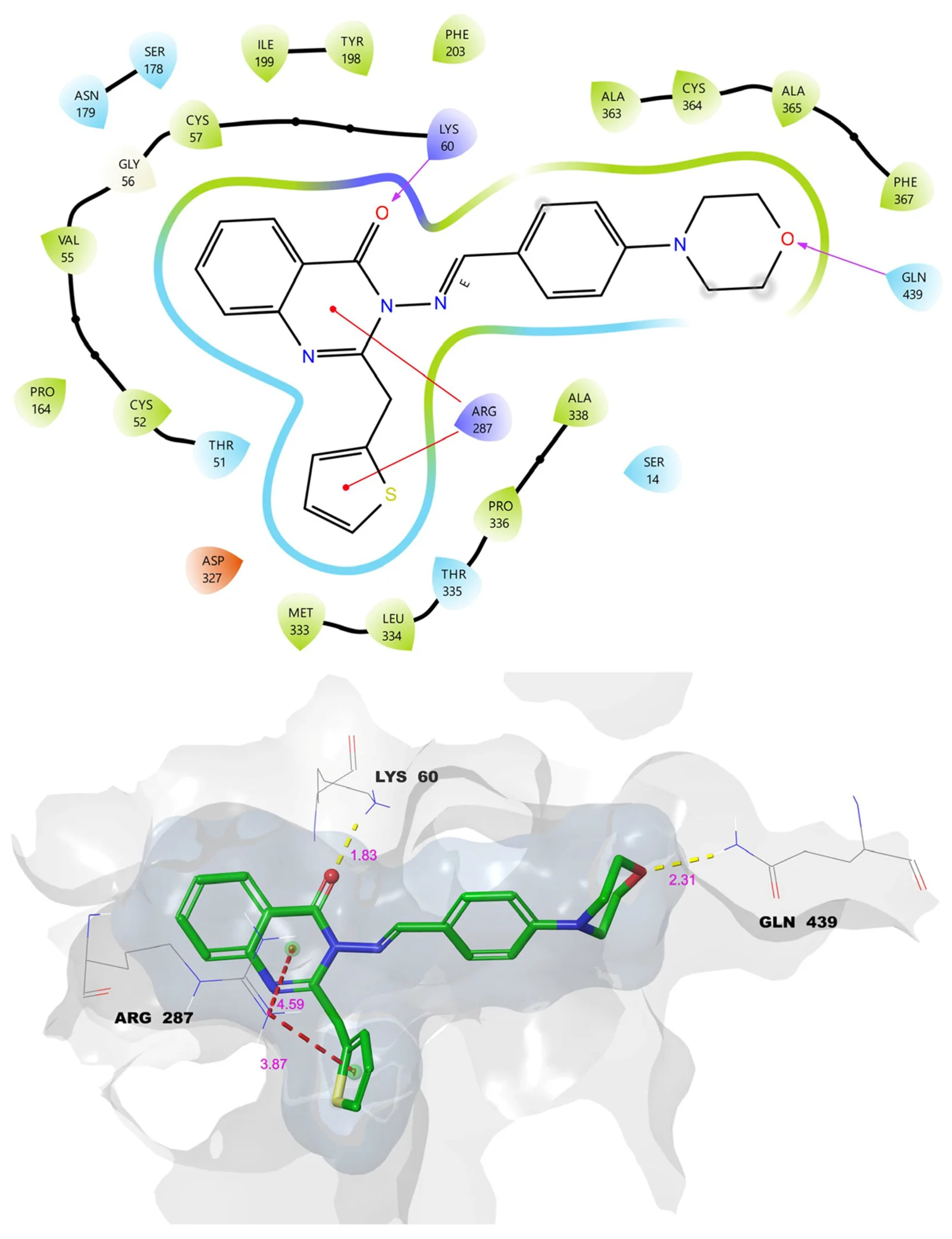
2D and 3D molecular docking interactions of the E9 –trypanothione reductase (TR) complex.

**Figure 4:**
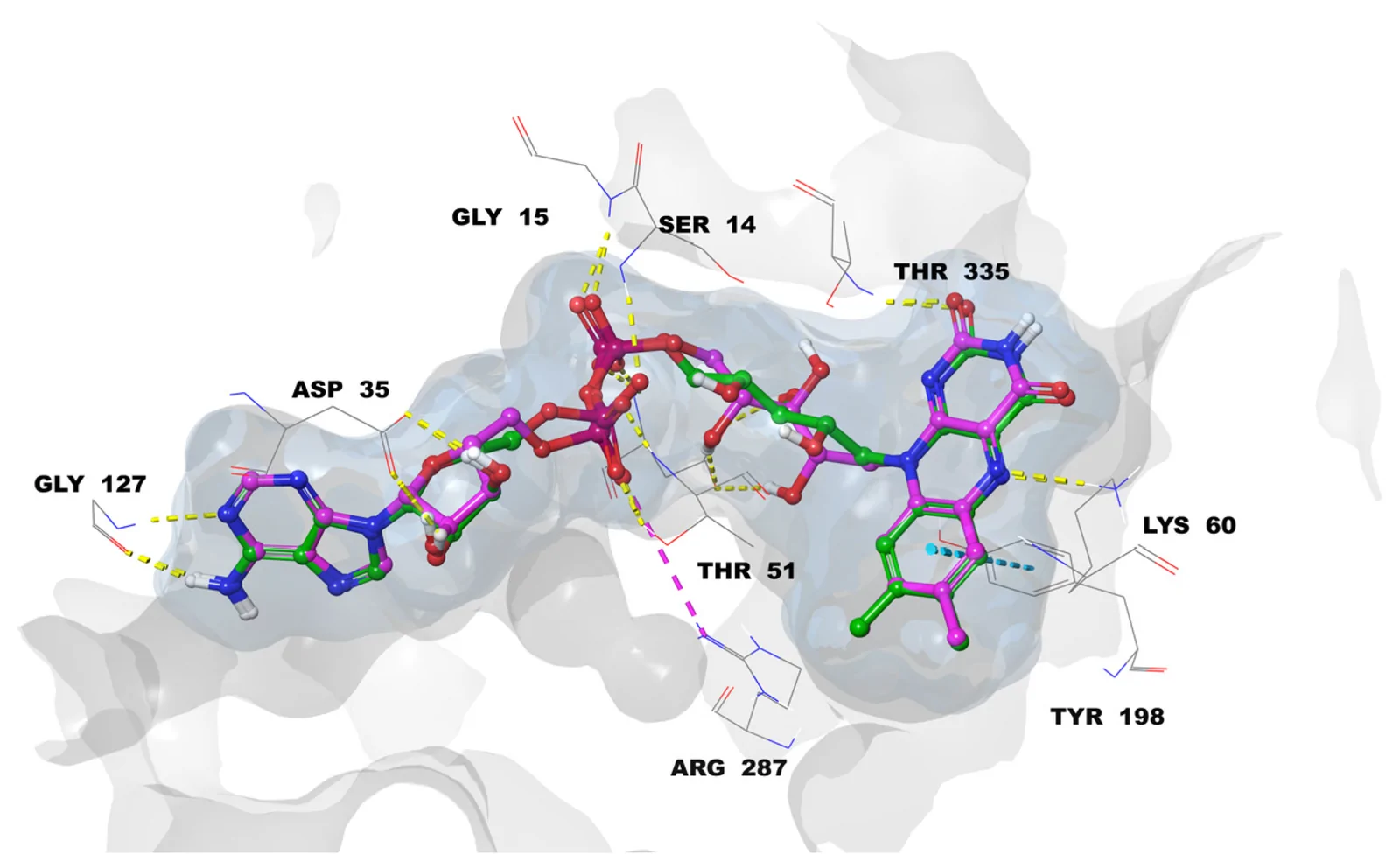
Redocking validation of the co-crystallized flavin adenine dinucleotide (FAD) ligand in the active site of trypanothione reductase (Protein Data Bank (PDB) ID: 2JK6). Native FAD is shown in green and redocked FAD in magenta; nitrogen and oxygen atoms are shown in blue and red, respectively.

**Figure 5:**
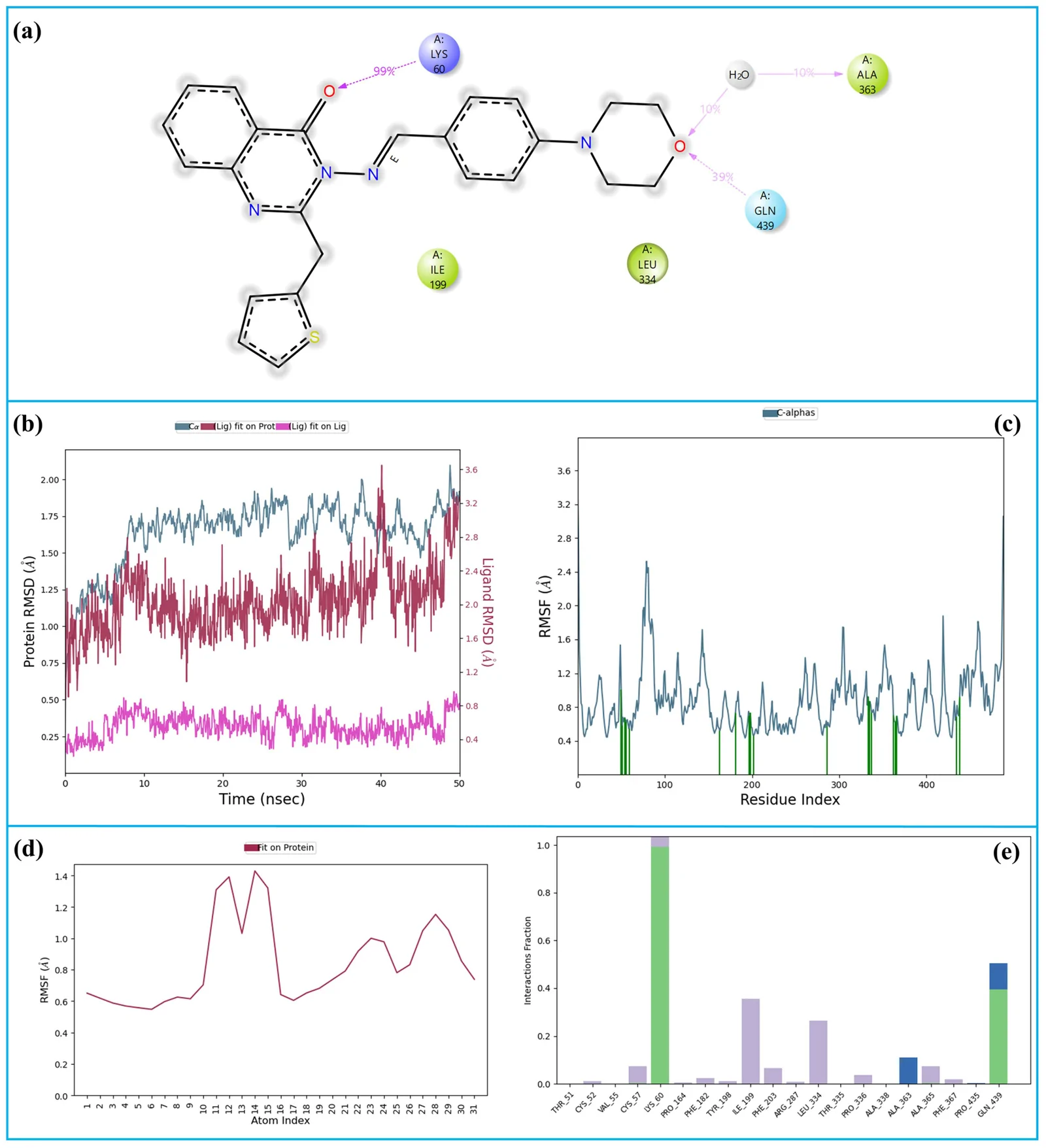
Results of the 50 ns molecular dynamics simulation of the E9—trypanothione reductase (TR) complex. (**a**) 2D map of dominant ligand–protein interactions; (**b**) time evolution of root-mean-square deviation (RMSD) for ligand and protein; (**c**) residue-wise root-mean-square fluctuation (RMSF) analysis of the protein; (**d**) RMSF profile of the ligand; (**e**) interaction fraction histogram showing contact persistence during the simulation.

**Figure 6:**
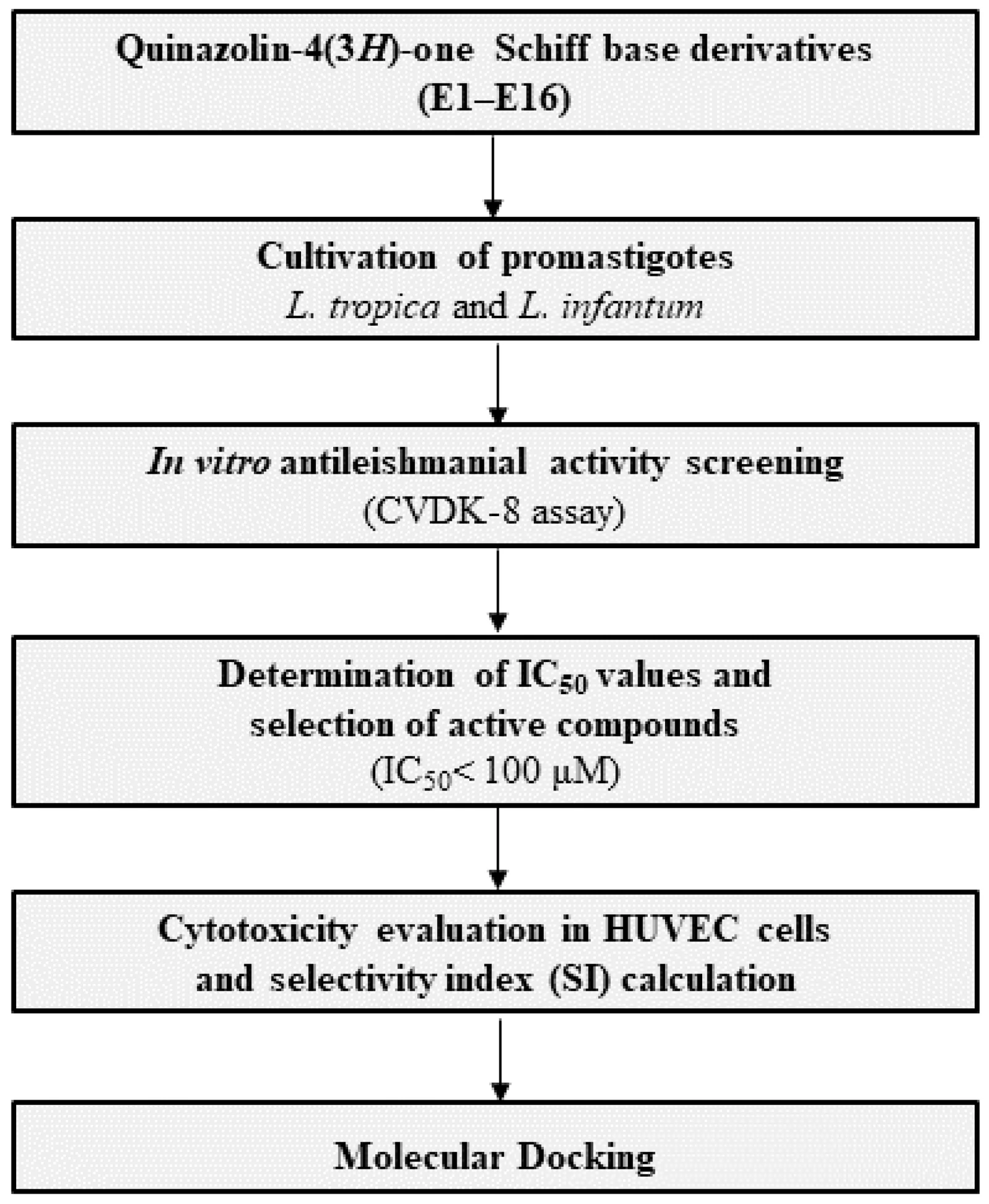
Schematic overview of the experimental workflow of the study.

**Figure 7:**
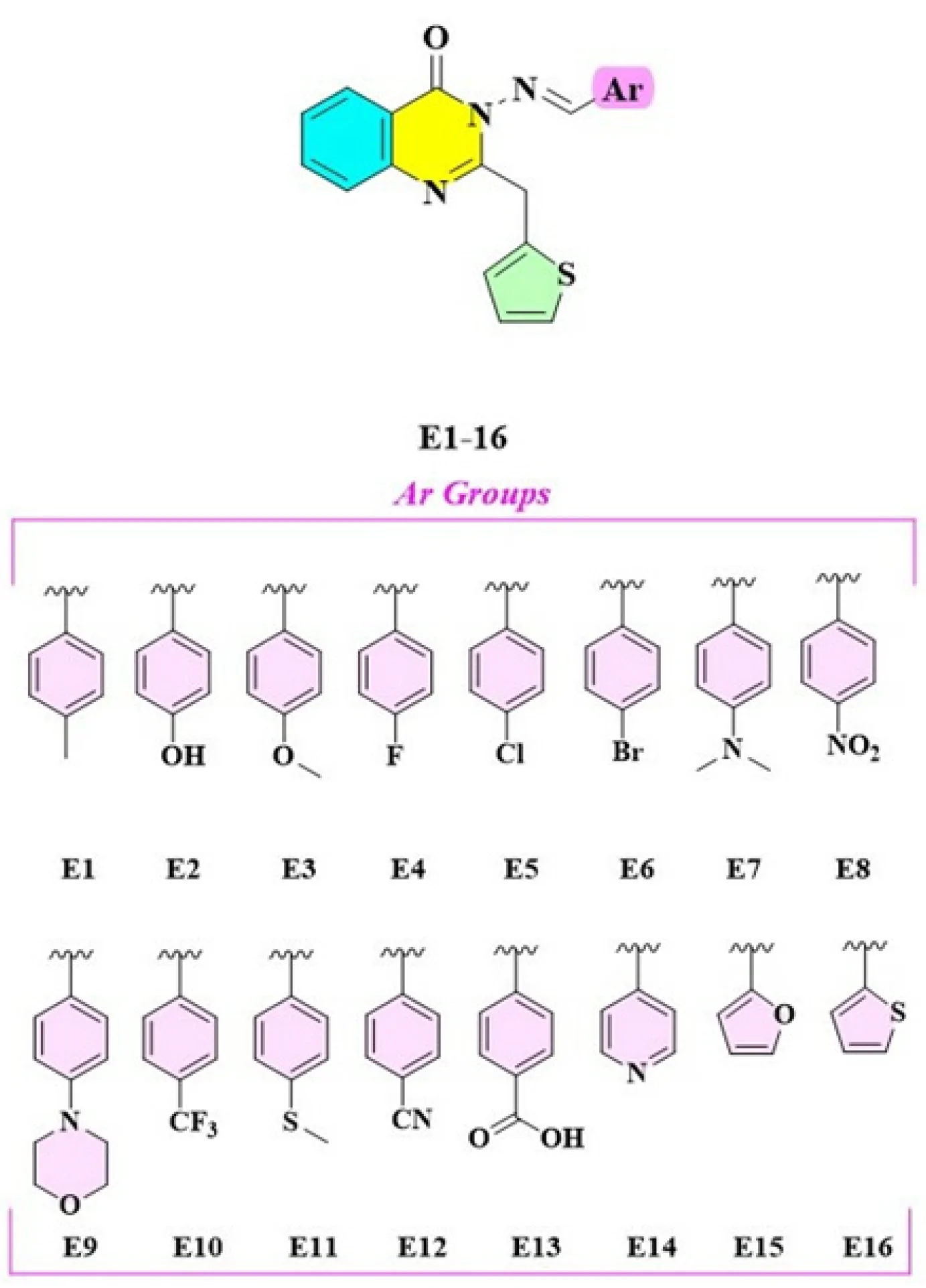
Quinazolin-4(3*H*)-one derivatives evaluated for their antileishmanial activities.

**Table 1:** In vitro cytotoxicity and selectivity indices of compounds E2, E3, E9, E11 and miltefosine.

Compound	IC_50_ (µM)			Selectivity Index (SI)	
	HUVEC	*L. infantum*	*L. tropica*	SI (*L. infantum*)	SI (*L. tropica*)
**E2**	205.3	38.1	23.2	5.4	8.8
**E3**	84.6	69.7	–	1.2	–
**E9**	17.2	28.2	22.4	0.6	0.8
**E11**	133.1	86.7	76.0	1.5	1.8
**Miltefosine**	7.6	33.8	21.8	0.2	0.3

**Table 2:** Chemical names of the quinazolin-4(3*H*)-one derivatives (E1–E16) evaluated in the present study.

Code	Chemical Name	mp (°C)	Color and State
**E1**	3-(4-Methylbenzylideneamino)-2-(thiophen-2-ylmethyl)quinazolin-4(3*H*)-one	155–157	White solid
**E2**	3-(4-Hydroxybenzylideneamino)-2-(thiophen-2-ylmethyl)quinazolin-4(3*H*)-one	220–222	White solid
**E3**	3-(4-Methoxybenzylideneamino)-2-(thiophen-2-ylmethyl)quinazolin-4(3*H*)-one	128–130	White solid
**E4**	3-(4-Fluorobenzylideneamino)-2-(thiophen-2-ylmethyl)quinazolin-4(3*H*)-one	154–156	Yellow solid
**E5**	3-(4-Chlorobenzylideneamino)-2-(thiophen-2-ylmethyl)quinazolin-4(3*H*)-one	158–160	White solid
**E6**	3-(4-Bromobenzylideneamino)-2-(thiophen-2-ylmethyl)quinazolin-4(3*H*)-one	157–159	Beige solid
**E7**	3-(4-Dimethylaminobenzylideneamino)-2-(thiophen-2-ylmethyl)quinazolin-4(3*H*)-one	180–182	Yellow solid
**E8**	3-(4-Nitrobenzylideneamino)-2-(thiophen-2-ylmethyl)quinazolin-4(3*H*)-one	197–199	Yellow solid
**E9**	3-(4-Morpholinobenzylideneamino)-2-(thiophen-2-ylmethyl)quinazolin-4(3*H*)-one	186–188	Beige solid
**E10**	3-(4-Trifluoromethylbenzylideneamino)-2-(thiophen-2-ylmethyl)quinazolin-4(3*H*)-one	152–154	White crystals
**E11**	3-(4-Methylthiobenzylideneamino)-2-(thiophen-2-ylmethyl)quinazolin-4(3*H*)-one	150–152	Yellow solid
**E12**	3-(4-Cyanobenzylideneamino)-2-(thiophen-2-ylmethyl)quinazolin-4(3*H*)-one	189–191	Beige crystals
**E13**	3-(4-Carboxybenzylideneamino)-2-(thiophen-2-ylmethyl)quinazolin-4(3*H*)-one	283–285	White solid
**E14**	3-(Pyridin-4-ylmethyleneamino)-2-(thiophen-2-ylmethyl)quinazolin-4(3*H*)-one	176–178	Brown crystals
**E15**	3-(Furan-2-ylmethyleneamino)-2-(thiophen-2-ylmethyl)quinazolin-4(3*H*)-one	124–126	White solid
**E16**	3-(Thiophene-2-ylmethyleneamino)-2-(thiophen-2-ylmethyl)quinazolin-4(3*H*)-one	156–158	White solid

## Data Availability

The original contributions presented in this study are included in the article. Further inquiries can be directed to the corresponding author.

## Abbreviations

The following abbreviations are used in this manuscript:

HUVEC	Human Umbilical Vein Endothelial Cell
NNN	Novy–MacNeal–Nicolle
FBS	Fetal Bovine Serum
DMEM	Dulbecco’s Modified Eagle Medium
MM/GBSA	Molecular Mechanics/Generalized Born Surface Area
RMSD	Root Mean Square Deviation
RMSF	Root Mean Square Fluctuation
TR	Trypanothione Reductase
FAD	Flavin Adenine Dinucleotide
